# Performance evaluation of hybrid maize (*Zea mays* L.) in Burkina Faso, sub-Saharan region of Africa

**DOI:** 10.1016/j.heliyon.2024.e38133

**Published:** 2024-09-19

**Authors:** Lardia Ali Bougma, Mahamadi Hamed Ouédraogo, Dominique Nikiéma, Mahamadou Sawadogo

**Affiliations:** Department of Plant Biology and Physiology, Biosciences Laboratory, Program Genetics and Plant Breeding, University Joseph Ki-Zerbo, Faculty of Life and Earth Sciences, BP:7021, Ouagadougou 03, Burkina Faso

**Keywords:** Maize, Hybrids, Agronomy, Genetic, Heritability, Heredity, Environments, Burkina Faso

## Abstract

Maize (*Zea mays* L.) is a staple food for many people in Burkina Faso. The cultivation of maize hybrid genotypes plays a crucial role in increasing maize production and productivity. Feeding the growing population of the country, expected to reach thirty million by 2035, using hybrid genotypes of maize is a challenge. The objective of this study was to identify the hybrid maize genotypes having a best adaptability in the agro-ecological context of Burkina Faso. Nine (09) hybrid maize genotypes were evaluated during the 2018/2019 cropping season, in nine locations of the country characterized by a rainfall varying between 800 and 1200 mm. The experimental design was a Randomized Complete Block Design (RCBD) with three replications. The results showed that grain yield of the hybrids varied depending on the genotype nature and the cropping environment. The use of hybrid maize significantly increased the grain yield per hectare in maize production. Among the tested hybrid maize genotypes, SD1 (9.054 tons ha^−1^), SD3 (7.683 tons ha^−1^), and SD6 (9.385 tons ha^−1^) significantly presented higher yields. Based on the grain yield, the best growing environments of hybrid maize are NEBOUM1, BAMA and SOUNGALODAGO. The best genotypes for most of the environments are the hybrids of pure line varieties. The heritability was more than 80 % for all the studied yield traits.

## 1 Introduction

Rainfed agricultural products account for more than 95 % of the agricultural productions in sub-Saharan Africa [1], and the main rainfed cereal crops grown are maize, millet, sorghum, and wheat. Yield gaps of smallholders' productions in sub-Saharan Africa (SSA) are among the largest in the world (https://www.yieldgap.org). While the demand in cereals is projected to double in the next decades, yields increases trends in cereals productions remain very slow [2]. In recent years, maize yields on a regional basis have ranged from 1 to 1.2 tons per hectare in West and Central Africa (https://www.yieldgap.org/). Demographic projections show that Africa's population will grow faster than any other continent in the coming years. Therefore, to meet the food security of populations, there is an urgent need to increase agricultural productions. The effectiveness of hybrid varieties in increasing yields have been proven and there is a particular interest of their use around the world. Results of the last ten years showed that in Europe and United States, a yield of 8 t ha^−1^ have been registered using cereals hybrid varieties [3]. Since 1950s, maize yields increase progresses per year in U.S. Corn Belt. Yield increases can be attributed to genetic progress through heterosis playing a central role [4]. During the 1904 year, the theory of hybrids impact on agriculture has been demonstrated by Shull [[5], [6], [7]]. Even under unfavorable production conditions, hybrid varieties show better agronomic performance than the other types of varieties [[8], [9], [10]]. The development of hybrids genotypes appears to be the best method in plant breeding for increasing yields [11]. The hybrid maize genotypes yield exceeds those of the parental lines of maize of 150 %–300 % [12,13]. Nowadays, one-way hybrids, three-way hybrids and double-way hybrids are created through breeding programs [14,15]. Through the findings of Mather, Kempthorne, and Falconer, quantitative genetic became widely known all over the world and contributed immensely to the improvement of plants and animals in the 20th century. Hybrids genetic gain results from their vigor, yield stability, their uniformity and improved resistance to stress. Maize is one of cereal crops that expressing better heterosis [[16], [17], [18]].

Controversial debates over the political acceptance of hybrids in SSA have set back the goals of plant breeding regarding the development of hybrid crops. However, in Burkina Faso, maize production area is about 9 % of the total agricultural area [19], and regarding the increasing demand in maize, hybrid maize varieties have been developed by research programs. Likewise, companies such as "SEMAFORT" introduced hybrid varieties in the country. Unfortunately, these hybrids have not been sufficiently evaluated for their stability and yield. Therefore, a multi-location assessment of these hybrid materials is necessary. Hence, this study aiming at identifying the hybrid maize genotypes having a best adaptability in the agro-ecological context of Burkina Faso.

## 2 Materials and methods

### 2.1 Study area

**Fig. 1:**
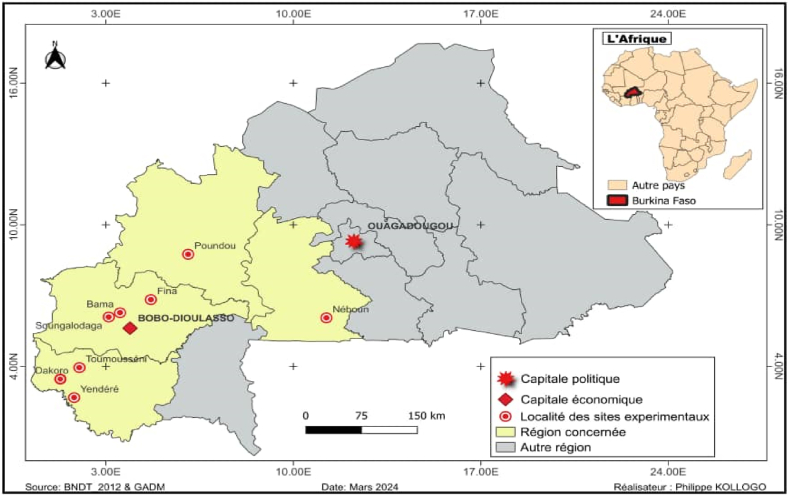
Geographical locations of experimental site.

The study was conducted in Burkina Faso, in nine (09) experimental sites, located in the western region of the country (Fig. 1). The country is located at 2°20′ N latitude and 2°20′ E and 50°3′ W longitude, with an area of 274,200 km^2^. Recorded weather information during the period of the experimentation of the different locations are presented in Table 1.

**Table 1:** Average maximal temperature and rainfall for the 10 research stations of the country for 2018 and 2019 years.

Station	Temp.		Rainfall		Annual number of rainy days		Relative humidity	
	2018	2019	2018	2019	2018	2019	2018	2019
Bobo-Dioulasso	33.7	36.0	1321	1402	100	96	72	72
Bogandé	36.0	36.0	562	534	54	63	56	54
Boromo	34.8	35.5	1024	1039	93	75	74	71
Dédougou	36.0	35.9	835	902	74	72	67	67
Dori	37	37.3	494	569	49	50	62	60
Fada N'Gourma	35.6	34.4	693	796	71	78	68	67
Gaoua	35.7	35.7	932	1235	100	94	79	78
Ouagadougou	35.6	36	860	853	80	79	66	64
Ouahigouya	35.3	34.8	927	800	54	72	57	58
Pô	34.8	34.6	1015	1005	82	90	74	72

### 2.2 Experimental design and plant maintenance

The experiment was conducted under rainfed conditions during the main cropping season of 2018/2019. The experimental design was a Randomized Complete Block Design (RCBD) with three replications. The gross plot size was of 0.80m × 0.40m. Six (06) hybrid maize genotypes collected from private seed Companies and three from the National Research institute were used for the study (Table 2). The hybrids were grown in each of the nine specific environments.

**Table 2:** Plant materials and their origin details.

Code	Designation	Genetic material	Reference	Country
SD1	WE3205	single cross multinational company	–	KENYA
SD2	WE4207	single cross multinational company	–	SOUTH AFRICA
SD3	SNK2778	double cross multinational company	[20]	NIGERIA/ZAMBIA
SD4	DK920	single cross multinational company	[20]	NIGERIA
SD5	DK234	single cross multinational company	[20]	NIGERIA
SD6	DK818	single cross multinational company	[20]	NIGERIA
SD7	SR21	hybrid national	[20]	BURKINA FASO
SD8	BONDOFA	hybrid national	[20]	BURKINA FASO
SD9	KOMSAYA	composite national	[20]	BURKINA FASO

Plant maintenance consisted of an organic and a mineral fertilization. The organic fertilizer (organic manure) was applied before ploughing the soil. The NPK (14:23:14: +6S) application was done 14 days after sowing. The urea (46 %) was implemented twice during plants developmental cycle. The first and the second applications were done at 25 and 45 days respectively.

### 2.3 Data collection

Eight agromorphological data were collected: plant height, ear height, number of rows per ear, number of grains per row, number of grains per ear, ear mean weight, grain moisture content and grain yield. The plant height was determined from the soil surface to the base of the tassel, while the ear height was determined from the soil surface to the base of the top ear. The yield was obtained using the yield components of the plants.

### 2.4 Statistical analyses

Genotype by environment interaction analysis was carried out using GenStat 9.2 version software. The heritability, the genotypic variance, the phenotypic variance and genotype x environment variances of the tested genotypes in the nine locations were estimated through six evaluated traits using formulas suggested by Falconer [21,22].

Heritability (broad sense), H^2^ = *σ*_*g*_^2^/*σ*_*p*_^2^, where *σ*_*p*_^2^ = phenotypic variance, and *σ*_*g*_^2^, = genotypic variance.

Phenotypic (*σ*_*p*_^2^) and genotypic (*σ*_*g*_^2^) variances were obtained using the method of [23] Baye, 2002 as follow: *σ*_*g*_^2^ = *MS*_p_-(*MS*_e_ ⁄*r*);*σ*_*p*_^2^ = *MS*_g_⁄*r*;*σ*_*e*_^2^ = *MS*_e_⁄*r*, where MS_p_, MS_g_ and MS_e_ are mean squares of phenotypes, genotypes, and of the error, respectively; r is the number of replicates.

The mean values were used for genetic analyses to determine the phenotypic coefficient of variation (PCV) and the genotypic coefficient of variation (GCV). According to Ref. [24] Singh and Chaudhury (1985):

GCV (%) = (√σ*g* ⁄ _X_) × 100, where x = sample mean.

PCV(%) = (√σ*p* ⁄ X) × 100, where x = sample mean.

Grain yield (tons ha^−1^) at 15 % moisture content was calculated using the following formula developed by Ref. [25]:

Grain yield tons ha^−1^ = PE (kg) x(100-H)/(100-Hr)x (Sx100)/(NPAx10)

where PE: weight of ear in kg, H: Grain moisture percentage at harvest; Hr: moisture content at 15 %; NPA: Net harvest plot area in m^2^; S: Shelling coefficient with 0.8.

XLSTAT 2016 version was used to perform the ANOVA and the means separation using Newman-Keuls (SNK) test. R package multienvironmental trials using the libraries ggplots2 (version 3.3.6) (https://cran.r-project.org/web/packages/ggplot2/ggplot2.pdf) [26] for the significance at individual location and over locations were carried out for each set of experiments. Critical differences were calculated at 5 % threshold.

## 3 Results and discussion

### 3.1 Estimation of the genetic parameters of the genotypes for the studied variables in each location

Table 3 shows the heritability, means, coefficients of variation (CV), phenotypic coefficients of variation (PCV) and genotypic coefficients of variation (GCV) for 7 parameters of the studied maize genotypes. Significant differences were observed between locations for all the genetic parameters of all the variables, except for ear height. The high ratio of heritability (H^2^), in each location expresses the extent to which hybrids' phenotypes are determined by the genotypes. Schnell [11] reported that the hybrids provide great stability under various environmental conditions. This is an essential aspect in the manifestation of heterosis in hybrids [27,28]. This characteristic of crops hybrid genotypes is important for farmers as it provides a certain production security. In all the locations involved in this study, the heritability has been more than 0.80 for the majority of the studied yield traits, except for FINA location. Significant variations of the genetic parameters were observed between the locations. This indicates that the environments greatly influenced the expression of the studied agro-morphological parameters. The significant differences of the phenotypic and genotypic coefficients of variation between locations for the grain yield showed that the environment plays a significant role in the inheritance of the characters. Authors as [21,22,29] reported that environmental variance is a source of error that reduces precision in genetic studies.

**Table 3:** Overall Mean performances, Heritability, Genotypic variance, Phenotypic variance and Coefficients of Variation.

	Statistic	ds	phe	ph	rn	en	rng	yld
Bama	**Heritability**	0,70	0,44	0,58	0,93	0,93	0,99	0,93
	**Genotypic variance**	25,12	0,04	0,07	6,59	1,36	4956,12	2,59
	**Phenotypic variance**	35,88	0,09	0,001	7,08	1,46	5006,18	2,78
	**Mean**	23,39	1,41	2,51	6,54	35,60	14,25	5,08
	**LSD (5 %)**	0,0001	0,0001	0,003	0,0001	0,0001	0,0001	0,0001
	**CV (%)**	14,10	15,58	8,66	3,18	3,83	2,33	11,74
Dakoro	**Heritability**	0,61	0,66	0,05	0,97	0,96	0,98	0,84
	**Genotypic variance**	12,79	0,02	0,01	7,22	1,60	5513,07	2,10
	**Phenotypic variance**	20,96	0,03	0,2	7,44	1,66	5625,58	2,5
	**Mean**	24,97	1,33	2,43	35,59	14,20	507,10	5,36
	**LSD (5 %)**	0,0001	0,719	0,246	0,0001	0,0001	0,0001	0,0008
	**CV (%)**	11,39	11,06	9,52	2,05	3,21	3,12	19,49
Fina	**Heritability**	0,77	0,30	0,66	0,97	0,96	0,99	0,74
	**Genotypic variance**	20,20	0,003	0,06	8,21	2,15	7275,98	1,74
	**Phenotype variance**	26,23	0,01	0,09	8,46	2,24	7349,47	2,35
	**Mean**	22,37	1,25	2,35	35,46	14,16	504,48	4,50
	**LSD (5 %)**	0,0001	0,196	0,003	0,0001	0,0001	0,0001	0,0118
	**CV (%)**	10,87	13,31	11,77	29,95	2,32	3,34	29,95
Neboum1	**Heritability**	0,91	0,3	0,83	0,91	0,98	0,99	0,91
	**Genotypic variance**	85,45	0,003	0,05	0,88	10,47	2,59	0,88
	**Phenotypic variance**	93,90	0,01	0,06	0,97	10,68	2,62	0,96
	**Mean**	27,93	1,37	2,50	5,63	35,98	14,01	5,63
	**LSD (5 %)**	0,0001	0,276	0,001	0,0001	0,0001	0,0001	0,0001
	**CV (%)**	17,54	10,80	8,57	9,15	1,58	2,13	9,15
Neboum 2	**Heritability**	0,56	0,5	0,83	0,97	0,95	0,99	0,85
	**Genotypic variance**	4,52	0,01	0,05	9,70	1,86	7481,64	1,21
	**Phenotypic variance**	8,07	0,02	0,06	10	1,96	7557,21	1,42
	**Mean**	23,16	1,23	2,34	35,64	14,01	501,62	5,21
	**LSD (5 %)**	0,039	0,004	0,0005	0,0001	0,0001	0,0001	0,0001
	**CV (%)**	14,12	10,49	8,93	2,47	3,61	2,07	15,33
Poundou	**Heritability**	0,78	0,5	0,67	0,91	0,93	0,97	0,91
	**Genotypic variance**	5,79	0,01	0,04	7,91	1,50	5509,24	0,74
	**Phenotypic variance**	7,42	0,02	0,06	8,69	1,61	5679,63	0,81
	**Mean**	19,64	1,16	2,21	35,14	13,98	492,70	2,29
	**LSD (5 %)**	0,0004	0,136	0,011	0,0001	0,0001	0,0001	0,0001
	**CV (%)**	11,30	16,36	10,99	3,58	3,81	4,33	20,43
Soungalodga	**Heritability**	0,70	0,37	0,66	0,97	0,95	0,98	0,93
	**Genotypic variance**	6,73	0,006	0,04	8,46	2,35	7041,08	2,90
	**Phenotypic variance**	9,61	0,016	0,06	8,72	2,47	7184,77	3,11
	**Mean**	25,14	1,37	2,56	35,45	14,18	505,46	7,06
	**LSD (5 %)**	0,004	0,0589	0,0117	0,0001	0,0001	0,0001	0,0001
	**CV (%)**	11,77	11,85	9,87	2,36	4,21	3,45	10,90
Toumousseni	**Heritability**	0,86	0,60	0,30	0,95	0,95	0,98	0,91
	**Genotypic variance**	17,32	0,006	0,006	9,02	2,61	8246,61	1,15
	**Phenotypic variance**	20,13	0,01	0,02	9,49	2,75	8414,91	1,26
	**Mean**	26,53	1,10	2,07	35,42	19,19	504,82	9,92
	**LSD (5 %)**	0,0001	0,0498	0,1525	0,0001	0,0001	0,0001	0,0001
	**CV (%)**	10,87	11,33	9,38	3,07	4,05	3,17	11,43
Yendere	**Heritability**	0,70	0,33	0,40	0,96	0,93	0,99	0,95
	**Genotypic variance**	6,97	0,01	0,02	8,09	1,52	6013,56	3,75
	**Phenotypic variance**	9,95	0,03	0,05	8,43	1,63	6074,30	3,95
	**Mean**	21,66	1,31	2,51	35,42	13,99	497,52	5,01
	**LSD (5 %)**	0,0009	0,0623	0,0692	0,0001	0,0001	0,0001	0,0001
	**CV (%)**	12,04	13,41	10,68	2,95	4,01	2,60	14,77

Note: LSD = Least significant difference; ds = stem diameter (mm); phe = insertion ear height on stem (m); ph = height plant (m); rn = number of rows on ear; en = number of grains on ear; rng = number grain per row; yld = yield (tons ha^−1^).

### 3.2 Interaction between genotypes and environments (GxE) on grain yield and grain yield performance according to the location

**Fig. 2:**
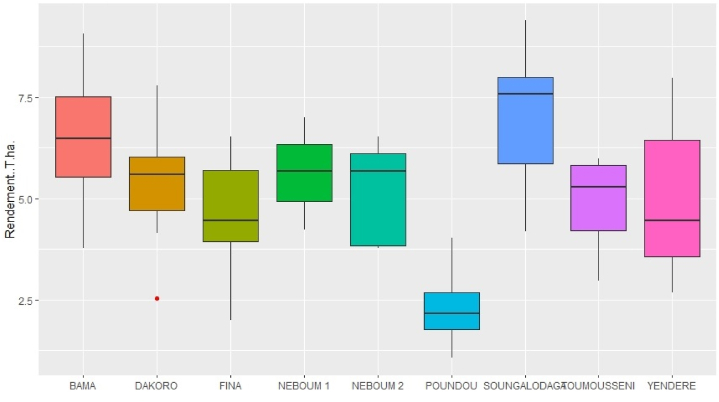
Performance in grain yield (tons ha^−1^) of hybrids per location.

Interactions between genotypes and environments on grain yield and the yield performance according to the environmental sites are presented in Table 4 and Fig. 2 respectively. The locations significantly affected the grain yield. As suggested by Falconer and Mackay [21,22], these observed differences reveal that a given genotype may be more performant to another genotype in a given environment and inversely in another. In this study, the registered average yields are not simply attributable to the genotype and the environment (P = G + E); they also result from an interaction component (I_GE_). This shows that the phenotype is the expression of a genotype placed in a given environment (P = G + E + I_GE_). The environment and the genotype are determinant factors in hybrids productivity. Indeed [2,3], showed that there is a good correlation between yield and environments in crop production. The grain yield of each maize hybrid genotype used varied from a specific environment to another. These variations of yields are results of interaction effects. The results showed as well that some hybrid genotypes were more sensitive to changing of environment compared to others. As depicted by Refs. [30,31], the results of this study suggest that the degree of the heterosis value does not only result from the genetic expression of a specific trait; it also depends on the environment where the character is measured. According to Refs. [32,33], an agromorphological parameter measured in two different environments is to be considered as two different parameters. This, because physiological mechanisms vary from an environment to another.

**Table 4:** Results of the Newman Keuls's Test for yield of the genotypes.

Var	NEB1	NEB2	TM	BAM	SG	FN	DAK	YEN	PD
**SD1**	6,992^**c**^	6,524^**b**^	5,794^**c**^	9,054^**d**^	8,714^**d**^	6,522^**c**^	7,788^**c**^	7,971^**d**^	4,016^**d**^
**SD2**	5,344^**ab**^	6,063^**b**^	5,982^**c**^	6,488^**b**^	6,081^**bc**^	4,315^**abc**^	5,868^**bc**^	4,134^**ab**^	2,096^**abc**^
**SD3**	5,027^**ab**^	5,724^**ab**^	4,324^**b**^	5,521^**b**^	7,683^**cd**^	5,648^**bc**^	4,900^**b**^	6,063^**c**^	2,932^**c**^
**SD4**	6,221^**bc**^	5,644^**ab**^	5,816^**c**^	7,249^**bc**^	7,734^**cd**^	6,480^**bc**^	6,480^**bc**^	4,775^**b**^	1,591^**ab**^
**SD5**	6,652^**c**^	6,283^**b**^	5,854^**c**^	6,451^**b**^	7,462^**cd**^	4,333^**abc**^	5,557^**bc**^	7,543^**d**^	2,584^**bc**^
**SD6**	5,987^**bc**^	3,853^**a**^	4,766^**bc**^	8,272^**cd**^	9,385^**d**^	5,636^**bc**^	5,636^**bc**^	3,217^**ab**^	1,829^**abc**^
**SD7**	4,237^**a**^	3,777^**a**^	3,893^**ab**^	5,543^**b**^	4,191^**a**^	2,004^**a**^	–	3,681^**ab**^	1,060^**a**^
**SD8**	–	–	–	3,774^**a**^	–	–	2,528^**a**^	–	–
**SD9**	4,626^**a**^	3,797^**a**^	2,960^**a**^	–	5,227^**ab**^	2,786^**ab**^	4,137^**ab**^	2,675^**a**^	2,241^**bc**^

Note: Means with same letter in column are not significantly different at *p = 0.05*,Hybrids of pure line: SD1:WE3205, SD2:WE4207, SD4:DK920, SD5:DK324, SD6:DK818.

Varieties for control: SD8: Bondofa, SD7: SR21, SD9: Komsaya.

Locations: NEB=NEBOUM; TM = TOUMOUSSENI; SG=SOUNGALODAGA; BAM=BAMA; FN=FINA; DAK = DAKORO; YEN=YENDERE; PD=POUNDOU.

### 3.3 Evaluation of genotypes stability

**Fig. 3:**
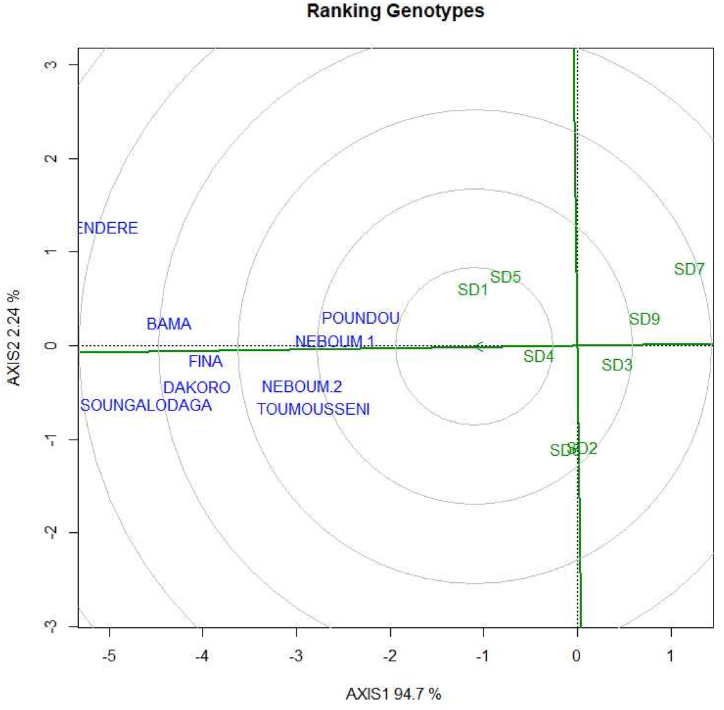
Ranking of the hybrids according to their stability using ggplot2 method.

The stability of maize hybrid genotypes was estimated by comparing the grain yield of the genotypes depending on the locations. A significant difference in stability was observed between the studied maize hybrid genotypes (Fig. 3). The pure lines' hybrids from single cross presented a greater stability compared to the others. The best stable pure lines hybrids were SD1, SD4 and SD5. The high performance of the pure lines’ hybrid noted are in line with the findings of [34,35], who reported that the heterosis was high in hybrids from single cross.

### 3.4 Classification of environments

**Fig. 4:**
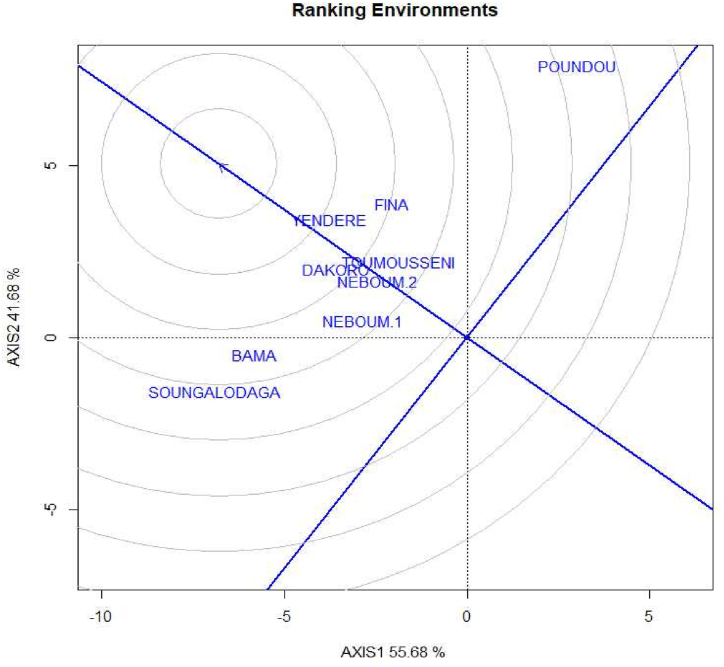
Ranking of the environments using ggplot2 method.

The results reveal a significant difference between the environments based on the grain yield of the maize hybrid genotypes (Fig. 4). Many of the locations have been favorable to the production of the studied maize hybrid genotypes. However, the best production sites were BAMA and SOUNGALODAGA locations. The lowest grain yields were registered from POUNDOU location. The interaction genotype by environment on the grain yield was significant for a greater number of the genotypes. The yields variations depending on the environments corroborate with [22] who showed that an environment can have increasing or decreasing effects on the stability a character.

## 4 Conclusion

Hybrids maize genotypes give higher grain yield. The heritability has been high for all the studied parameters. The current results indicate that all the experimental locations are appropriate for hybrids maize production, except for FINA and POUNDOU locations. The pure line hybrids from single cross showed the highest grain yield. The interaction between genotype and environment (G x E x I) had a significant effect on genotypes performance. The best stable pure lines hybrids were SD1, SD4 and SD5. The best production sites were BAMA and SOUNGALODAGA locations. Our results support the hypothesis suggesting that the hybrids from pure line have greater performance independently of the location. The use of the best identified hybrids in this study can considerably contribute to enhancing maize production in Burkina Faso.

## Data availability statement

Data will be made available on request.

## Additional information

No additional information is available for this paper.

## CRediT authorship contribution statement

**Lardia Ali Bougma:** Methodology, Formal analysis, Conceptualization. **Mahamadi Hamed Ouédraogo:** Writing – original draft, Validation, Funding acquisition. **Dominique Nikiéma:** Software, Formal analysis, Data curation. **Mahamadou Sawadogo:** Writing – original draft, Supervision.

## Declaration of competing interest

The authors declare the following financial interests/personal relationships which may be considered as potential competing interests:Bougma Lardia Ali reports a relationship with Joseph Ki-Zerbo University that includes: employment. Co-author previously employed by Joseph Ki-Zerbo University If there are other authors, they declare that they have no known competing financial interests or personal relationships that could have appeared to influence the work reported in this paper.
